# Coexistence of P190 and P210 BCR/ABL transcripts in chronic myeloid leukemia blast crisis resistant to imatinib

**DOI:** 10.1186/s40064-015-0930-x

**Published:** 2015-04-09

**Authors:** Zhao Junmei, Yu Fengkuan, Song Yongping, Fang Baijun, Liu Yuzhang, Liu Lina, Zhang Qinglan

**Affiliations:** Henan Key Lab of Experimental Haematology, Henan Institute of Haematology, Henan Tumor Hospital affiliated to Zhengzhou University, 127 Dongming Road, Zhengzhou, Henan Province 450008 China

**Keywords:** Chronic myeloid leukemia, BCR/ABL, Rsistance, Ematinib

## Abstract

**Introduction:**

Philadelphia chromosome (Ph) is a hallmark of chronic myeloid leukemia (CML), which exists in more than 90% CML and in 3% to 40% acute lymphoblastic leukemia (ALL).

**Case description:**

A 25-year-old man was diagnosed with CML in chronic phase. He first received treatment with hydroxyurea, achieving hematological remission and following imatinib mesylate for main treatment. A year later, he began to appear unexplained high fever with ineffective antibiotic treatment and bone morrow and blood tests indicated blast crisis. Both BCR/ABL 210 and BCR/ABL 190 fusion transcript were positive. Imatinib resistance was confirmed by a screening for ABL kinase domain E255K mutations, and dasatinib was administered. After two months, the patient went on to hematological remission.

**Discussion and evaluation:**

During medical treatment for CML, we experienced a relatively rare case with co-expression of the p210 and p190 encoding BCR-ABL transcripts in blastic phase. Imatinib resistance was confirmed and remission wasn’t easily obtained, yet dasatinib was helpful. When resistance emerges, the treatment options include increasing the daily dose of imatinib, or combining imatinib with other agents. Of course, dasatinib, nilotinib and bone marrow transplantation are good choice as well.

**Conclusions:**

The presence of p-190 transcript in CML may be related to progression of the disease. Thus monitoring the resistance of imatinib in CML patients, especially for advanced phase CML and BCR-ABL ALL, may be meaningful to guide clinical treatment and predict the prognosis.

## Introduction

Philadelphia chromosome (Ph) is a hallmark of chronic myeloid leukemia (CML), which exists in more than 90% CML and in 3% to 40% acute lymphoblastic leukemia (ALL) (Westbrook et al. 1992). It derives from a reciprocal translocation between chromosome 9 and 22, t (9; 22) (q34; q11), which results in the fusion of the 3' part of the ABL gene on chromosome 9 and the 5' part of the BCR gene on chromosome 22 (Bartram et al. 1983). In most Ph positive CML, the breakpoint in the BCR gene occurs in a small 5.8 Kb major breakpoint cluster region (M-bcr). This hybrid BCR-ABL gene in CML is transcribed into a novel 8.5 kb mRNA with a b3a2 and/or b2a2 junction. The mRNA encodes 210-kD fusion protein that can enhance protein tyrosine kinase activity. In the majority of Ph positive ALLs, the breakpoint is located in the first intron of the BCR gene known as the minor breakpoint cluster region (m-bcr).This fusion of BCR exon 1 to ABL exon 2 (ela2) leads to a 7.0-kb mRNA transcript that encodes the190-kD protein (Chissoe et al. 1995). Here, we report a patient co-expressed the p210 and p190 BCR-ABL transcripts in blast crisis.

Imatinib mesylate (IM), a new tyrosine kinase inhibitor, specifically targets BCR-ABL, which brings revolutionary era to the treatment of CML (Druker 2002, Savage and Antman 2002). Although the efficacy of IM is widely proved, resistance to IM has become a pressing challenge in the treatment of CML, especially in patients with advanced phases of the disease (Gambacorti-Passerini et al. 2003). The mechanisms of resistance have been described before, while the presence of ABL kinase domain is the most frequent mechanism of acquired resistance.

## Case report

**Figure 1:**
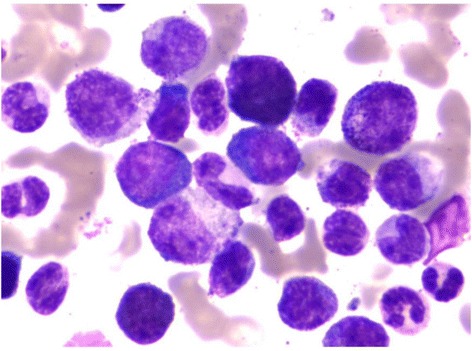
**Bone marrow examinations at initial diagnosis. (Wright-Giemsa staining, ×1000).**

**Figure 2:**
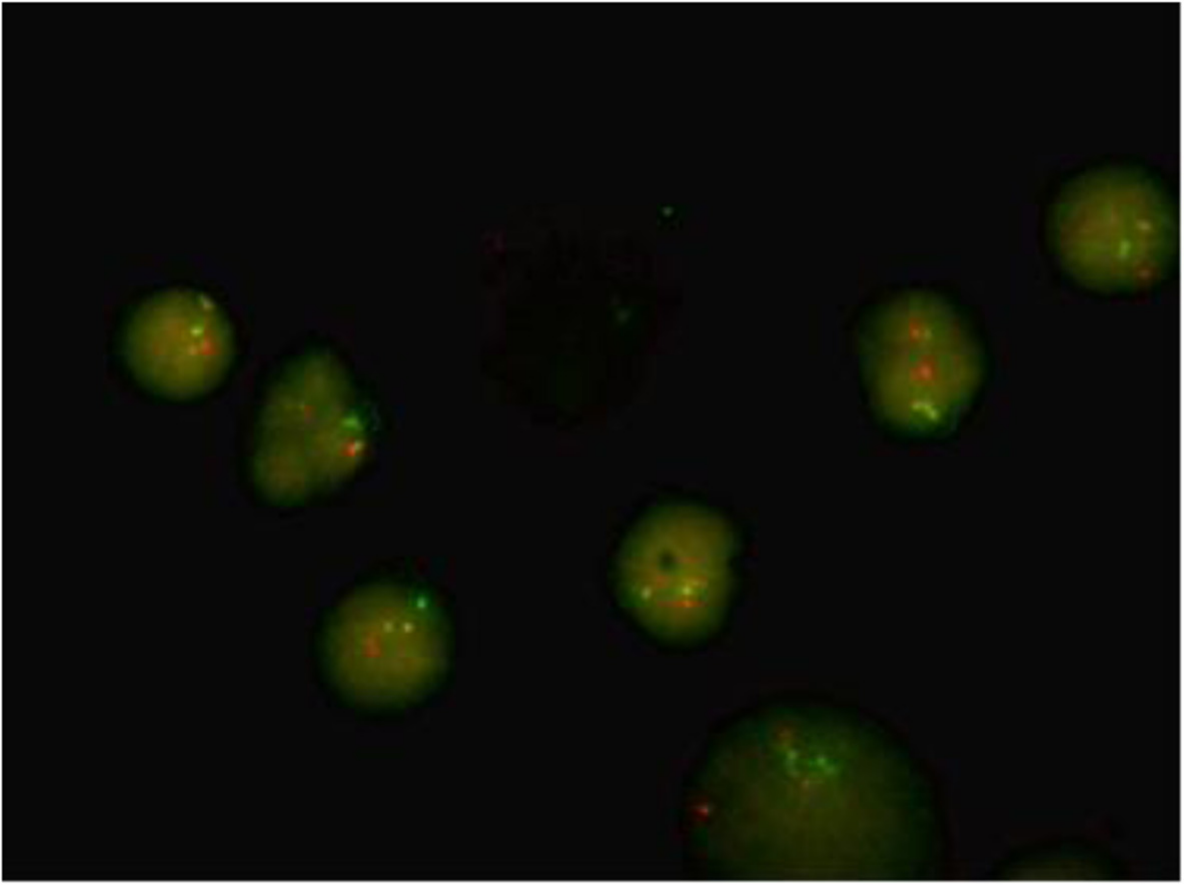
**Interphase cells from CML marrow at diagnosis showing ABL (red signal), BCR (green signal) and BCR-ABL fusion (yellow signal).**

A 25-year-old man was diagnosed as having a CML in December 2012. At diagnosis, physical examination revealed palpable spleen and liver. Laboratory tests showed leukocytosis(137 × 10^9^/L))with 2.5%myeloblasts,3.4%promyelocytes, 87.1%neutrophils, 6% eosinophils, and 1% basophils. The Hb was 11.3 g/dL and PLT 152 × 10^9^/L. Lactate dehydrogenase level was 2782 IU/dL. Bone marrow examination showed hypercellularity with myeloid hyperplasia with 2.4% myeloblasts, 2.8% promyelocytes, 20% myelocytes, 18% metamyelocytes, 6% eosinophils, 0% basophils (Figure 1). Peripheral blood smear and bone marrow trephine showed the typical features of chronic phase CML. Cytogenetic studies on marrow specimen showed a 46XY karyotype with *t*(9;22)(q34;q11) in all of 20 metaphase cells. Ph-positive ratio in bone marrow cells analyzed by fluorescence in situ hybridization (FISH) was 21.2% (Figure 2). The BCR/ABL 210 fusion transcript was found by reverse transcriptase polymerase chain reaction (RT-PCR) and BCR/ABL 190 fusion transcript wasn’t detected.

He was initially treated with hydroxyurea. About one month later, he went on to hematological remission. Then the patient was treated with imatinib mesylate (IM 400 mg, qd) for more than one year. At 3, 6, 9 and 12 months after diagnosis, BCR/ABL 210 fusion transcript detected by RT-PCR was still positive and BCR/ABL 190 fusion transcript was negative. In April 2014, he began to appear unexplained high fever with ineffective antibiotic treatment and a rise in WBC was detected. Bone marrow examination showed hypercellularity with myeloid hyperplasia with 25% myeloblasts. Flow cytometry analysis showed blasts accounted for 21.88% of the total cells and were positive for CD34, CD38, HLA-DR, CD13, CD33, CD117, CD9 and negative for MPO, cyCD3, cyCD79a, CD19, CD3, CD2, which suggested this patient entered into blast crisis (BC). Both BCR/ABL 210(0.83 BCRABL⁄ABL ratio) and BCR/ABL 190(0.001BCRABL⁄ABL ratio) fusion transcript were positive. He was treated with HA (HHA 4 mg d1-7,Ara-C 200 mg d1-7) and MA(MIT 10 mg d1-3, Ara-C 200 mg d1-7) regimen. However, hematological remission was not obtained. In June 2014, he was treated with HAG (HHA 2 mg d1-14, Ara-C 25 mg × 2 d1-14, G-CSF 300ug d1-14). Fortunately, he could achieve partial hematological remission after this induction chemotherapy and BCR/ABL 190 fusion transcript Gene turned to negative. Imatinib resistance was confirmed by a screening for ABL kinase domain E255K mutations, and dasatinib was administered. After two months, the patient went on to hematological remission.

## Discussion

It is generally thought that p210 BCR-ABL gene is observed in most Ph-positive CML patients, while the breakpoint in m-bcr is more frequently found in Ph-positive ALL and it is generally associated with an acute leukemia phenotype (Melo 1996). However, co-expression of the p210 and p190 encoding BCR-ABL transcripts is relatively rare. Some researchers indicated that the presence of p190 fusion gene was associated with a blastic phase as clinical presentation of the disease in most CML patients (Costello et al. 1995, Yamaguchi et al. 1998). In this case report, the patient co-expressed the p210 and p190 BCR-ABL transcripts in BC and remission wasn’t easily obtained, suggesting that the presence of the p-190 transcript in CML may be related to progression of the disease. However, it is worth wondering whether the presence of the p-190 transcript accounts for the resistance to imatinib treatment.

Several mechanisms that have been proposed may explain the resistance to imatinib (Agirre et al. 2003, Gambacorti-Passerini et al. 2000, Shah et al. 1998). Point mutations in the kinase domain of ABL are recognized as the major cause, especially in patients with advanced phase CML and BCR-ABL ALL.

Up to now, more than 50 mutants have been researched (Jabbour et al. 2006, Willis et al. 2005). Hochhaus et.al demonstrated that the substitutions of E255 resulted in virtual insensitivity to imatinib (Hochhaus et al. 2002). Other researchers discovered that ATP binding (P) loop, a highly conserved glycine rich sequence occupying residues 248-255 of ABL mutations, were related to a more rapid progression to advanced-phase disease than mutations in other regions (Soverini et al. 2005).

When resistance emerges, the treatment options include increasing the daily dose of imatinib, or combining imatinib with other agents. Of course, if economic conditions permit, more potent kinase inhibitors, such as dasatinib and nilotinib. and bone marrow transplantation are good choice as well. In this case, we choosed dasatinib as a treatment for imatinib resistance.

## Conclusions

We detailedly described a man who was co-expression of the p210 and p190 encoding BCR-ABL transcripts in CML BC. The presence of p-190 transcript in CML may be related to progression of the disease. Thus monitoring the resistance of imatinib in CML patients, especially for advanced phase CML and BCR-ABL ALL, may be meaningful to guide clinical treatment and predict the prognosis.
